# SuhB Associates with Nus Factors To Facilitate 30S Ribosome Biogenesis in *Escherichia coli*

**DOI:** 10.1128/mBio.00114-16

**Published:** 2016-03-15

**Authors:** Navjot Singh, Mikhail Bubunenko, Carol Smith, David M. Abbott, Anne M. Stringer, Ronald Shi, Donald L. Court, Joseph T. Wade

**Affiliations:** a Wadsworth Center, New York State Department of Health, Albany, New York, USA; b Frederick National Laboratory for Cancer Research, National Cancer Institute, Frederick, Maryland, USA; c Department of Biomedical Sciences, University at Albany, Albany, New York, USA

## Abstract

A complex of highly conserved proteins consisting of NusB, NusE, NusA, and NusG is required for robust expression of rRNA in *Escherichia coli*. This complex is proposed to prevent Rho-dependent transcription termination by a process known as “antitermination.” The mechanism of this antitermination in rRNA is poorly understood but requires association of NusB and NusE with a specific RNA sequence in rRNA known as BoxA. Here, we identify a novel member of the rRNA antitermination machinery: the inositol monophosphatase SuhB. We show that SuhB associates with elongating RNA polymerase (RNAP) at rRNA in a NusB-dependent manner. Although we show that SuhB is required for BoxA-mediated antitermination in a reporter system, our data indicate that the major function of the NusB/E/A/G/SuhB complex is not to prevent Rho-dependent termination of rRNA but rather to promote correct rRNA maturation. This occurs through formation of a SuhB-mediated loop between NusB/E/BoxA and RNAP/NusA/G. Thus, we have reassigned the function of these proteins at rRNA and identified another key player in this complex.

## IMPORTANCE

As RNA polymerase transcribes the rRNA operons in *E. coli*, it complexes with a set of proteins called Nus that confer enhanced rates of transcription elongation, correct folding of rRNA, and rRNA assembly with ribosomal proteins to generate a fully functional ribosome. Four Nus proteins were previously known, NusA, NusB, NusE, and NusG; here, we discover and describe a fifth, SuhB, that is an essential component of this complex. We demonstrate that the main function of this SuhB-containing complex is not to prevent premature transcription termination within the rRNA operon, as had been long claimed, but to enable rRNA maturation and a functional ribosome fully competent for translation.

## INTRODUCTION

Transcription termination in bacteria occurs by two distinct mechanisms: intrinsic (Rho independent) and Rho dependent. Intrinsic termination occurs without the need for factors other than the RNA polymerase (RNAP) and RNA (1). Rho-dependent termination requires a protein cofactor, Rho. Rho is an ATP-dependent RNA helicase that loads onto nascent RNA and translocates along the RNA in a 5′-to-3′ direction. Once Rho catches the elongating RNAP, it terminates transcription by promoting a rearrangement of the RNAP active site (2). Translation protects RNA from Rho-dependent termination (3), and therefore, noncoding RNAs have been considered likely candidates for Rho-dependent termination (4).

Modulation of transcription termination is a critical part of the life cycle of lambdoid bacteriophage. Early work on λ phage indicated that phage and host proteins combine to prevent both Rho-dependent and intrinsic termination within the phage genome. In particular, two phage-encoded proteins, N and Q, were identified as key antitermination factors (5). N-mediated antitermination occurs within the p_L_ and p_R_ transcripts, allowing RNAP to transcribe the early λ genes, including Q, which in turn allows late gene transcription. Specific RNA sequences, NutL and NutR, are required for N function (6, 7), as are host proteins NusB, NusE (ribosomal protein S10), NusA, and NusG, collectively known as Nus factors (8–13). The Nut sequences contain two important regions: BoxA and BoxB. BoxA serves as a binding site for a complex of NusB and NusE (14), and BoxB serves as a binding site for N (15). These proteins, together with the RNAP-associated elongation factors NusA and NusG, modify the RNAP such that it is resistant to both Rho-dependent and intrinsic termination (5, 15). *In vitro*, high levels of N obviate the requirement for NusB, NusE, and BoxA (16), suggesting that the role of BoxA-NusB/E complex is to stabilize the antitermination complex (17).

In addition to their role in the life cycle of bacteriophage, Nus factors are required in many bacterial species for proper expression of rRNA (18). rRNA loci in *Escherichia coli*, and many other species, contain two copies of *boxA* a short distance upstream of the 16S and 23S rRNA genes. Several independent observations have led to the suggestion that Nus factors prevent Rho-dependent termination within rRNA in a BoxA-dependent manner. First, Rho-dependent termination of a reporter construct is inhibited by insertion of sequence from the leader region of rRNA (19). This antitermination activity has been localized to a DNA segment containing a *boxA* sequence (20) and requires functional NusB and NusG (21). Second, *nusB* and *nusA* mutants exhibit polarity within the rRNA, having a significantly higher 16S:23S rRNA ratio (30S:50S) than wild-type cells (22, 23). Third, high-level transcription of RNA containing λ NutL titrates Nus factors, thereby decreasing rRNA expression and increasing the 16S:23S rRNA ratio (24). Fourth, mutation of *boxA* results in a significant reduction in rRNA synthesis from an rRNA operon carried on a plasmid (25, 26). Fifth, Rho-dependent transcription termination *in vitro* can be inhibited in a BoxA-dependent, NusB-dependent manner (27). BoxA and Nus factors also modulate RNAP such that it elongates at a higher rate and is resistant to the effects of ppGpp (28–30). The mechanism by which Nus factors prevent Rho-dependent termination is unclear, although it has been suggested that a NusE-NusG interaction can prevent association of Rho with RNAP-associated NusG, a critical requirement for Rho-dependent termination (31).

Although many studies have suggested a role for Nus factors in antitermination of rRNA, these proteins have recently been suggested to have an alternative role at rRNA: promoting correct folding and assembly of rRNA (22). The rRNA operon is transcribed as a single RNA that is then processed by several RNases to generate 16S, 23S, and 5S rRNAs, and also tRNAs (the tRNA complement differs for each of the seven copies of the rRNA locus in *E. coli*) (32). Mutants of *nusB* and *nusA* are defective in rRNA maturation, and accumulate 30S ribosome precursors (22). This property and the cold-sensitive growth phenotype of these mutants are suppressed by mutations in *rnc*, the gene encoding RNase III (22). Given that RNase III is not known to be involved in Rho termination, the genetic connection between *rnc* and *nus* genes suggests that the defects of *nus* mutants in rRNA maturation are unconnected to antitermination. Nus factors have been proposed to act as rRNA chaperones, promoting loop formation between NusB/E bound to BoxA and the elongating RNAP, thereby facilitating rRNA folding, ribosome protein assembly, and ribosome maturation (22). RNase III is responsible for the initial step in 16S and 23S rRNA processing. Mutations in *rnc* have been proposed to suppress the growth defects of Nus factor mutants by artificially stabilizing the stem-loop at the base of the 16S rRNA that is normally cleaved by RNase III (22).

SuhB is a widely conserved inositol monophosphatase (IMPase), and IMPase activity has been demonstrated for the *E. coli* enzyme *in vivo* and *in vitro* (33). However, myo-inositol-containing phospholipids and soluble inositol compounds are not detectable in *E. coli*, strongly suggesting that IMPase activity is not its primary function (34). Consistent with this, mutants of *E. coli* *suhB* have several characteristics that suggest a function for SuhB beyond its enzymatic activity. First, cells lacking *suhB* (also known as *ssyA*) are cold sensitive, but the growth defect is not associated with SuhB mutants defective in IMPase activity (35). Second, mutants of *suhB* suppress the growth defects of a *secY* mutant (36). *secY* mutants have a reduced rate of protein translocation across the cytoplasmic membrane, and suppressors have been proposed to have translation defects that disrupt the coordination of translation and secretion (36–39). Third, the cold sensitivity of *suhB* mutants is suppressed by mutations in *rnc* (40). These phenotypes suggest a connection to NusB, since *nusB* mutants are also defective in translation (23), can suppress a *secY* mutant growth defect (39, 41) and are themselves cold sensitive and are suppressed by mutations in *rnc* (22). Moreover, SuhB has been shown to interact with RNAP *in vitro* (35).

Although rRNA antitermination can be reconstituted *in vitro* with cell extracts, complete rRNA antitermination cannot be achieved using purified Nus factors alone (27). The efficiency of antitermination can be increased by inclusion of ribosomal protein S4, but S4 antagonizes Rho independently of BoxA and is insufficient to provide complete antitermination (42). Hence, it has been suggested that at least one member of the rRNA antitermination machinery is yet to be discovered (27). Here, we show that SuhB is such a protein. SuhB associates with elongating RNAP at rRNA loci in a NusB-dependent manner. Moreover, SuhB, like NusB, was required for antitermination in an *in vivo* reporter gene assay. Surprisingly, our data indicate that SuhB and Nus factors are largely dispensable for rRNA antitermination *in vivo* and that rRNA is quite resistant to Rho-dependent termination. Rather, our data support a role for SuhB in rRNA maturation and suggest a model in which SuhB promotes loop formation between elongating RNAP and NusB/E bound to BoxA.

## RESULTS

### SuhB is functionally connected to Nus factors.

To investigate the function of SuhB, we isolated five spontaneous mutants that suppress the cold sensitivity of *suhB* deletion in *E. coli* MG1655. Mutation of *rnc* has previously been reported to suppress the growth defect of a *suhB* mutant (40). Hence, we first PCR amplified and sequenced *rnc* from each suppressor mutant and identified four mutations in *rnc* (see Table S3 in the supplemental material). We then sequenced the genome of a suppressor mutant that had wild-type *rnc* and thereby identified a mutation in *nusE* (corresponding amino acid change, L17Q [see Table S3]). No other mutations were identified in this strain. To confirm the importance of the *nusE* mutation in suppressing the cold sensitivity of *suhB* deletion, we P1 transduced a *nusE*-linked *tetA* gene (conferring tetracycline resistance) from a strain with wild-type *nusE* into the Δ*suhB* strain with the *nusE* mutation and selected for growth at 42°C on medium containing tetracycline. The *tetA* gene is predicted to cotransduce with *nusE* ~44% of the time (43). A total of 55 of 98 transductants tested were cold sensitive. We PCR amplified and sequenced *nusE* from 10 colonies that were cold sensitive and 10 that were not. All the cold-sensitive strains had a wild-type copy of *nusE*, whereas all the cold-resistant strains had retained the mutant copy. We conclude that the *nusE* mutation is necessary for suppression of the cold sensitivity caused by deletion of *suhB*.

**FIG 1:**
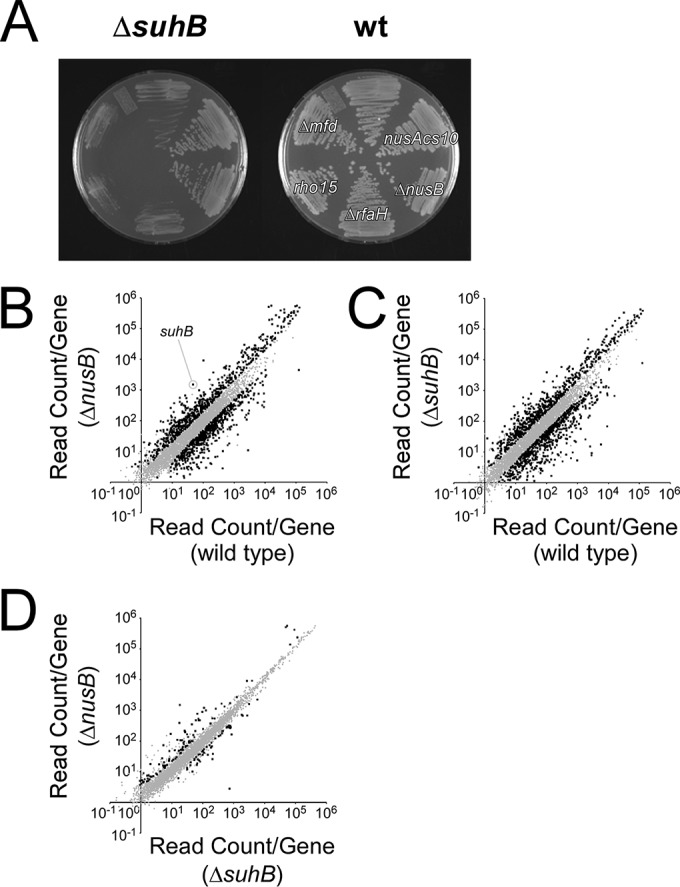
SuhB is functionally related to Nus factors. (A) Growth phenotypes of single mutants of *mfd*, *nusA*, *nusB*, *rho*, and *rfaH* in the W3110 wild-type (wt) background compared to the phenotypes for strains with the same mutations combined with deletion of *suhB*. Cells were restreaked onto LB medium and grown overnight at 37°C. (B) RNA-seq comparison of wild-type MG1655 and an isogenic Δ*nusB* strain. Each dot represents an annotated gene. Values on each axis indicate the relative number of sequence reads mapping to a given gene (plotted on a log scale), with values for the wild type plotted on the *x* axis and values for the Δ*nusB* mutant plotted on the *y* axis. Data points shown in black indicate genes for which we detected a significant difference (FDR, <0.05) of >2-fold between the wild-type and Δ*nusB* strain. (C and D) Equivalent comparisons for wild-type and Δ*suhB* strains (C) and Δ*nusB* and Δ*suhB* strains (D).

Identification of a *nusE* mutant suppressor directly connects SuhB function to that of the Nus factors. To further investigate the connection between SuhB and Nus factors, we constructed derivatives of Δ*suhB* W3110 with additional mutations in each of *nusA*, *nusB*, *rho*, and two other genes, *mfd* and *rfaH*, that encode RNAP-associated proteins. Only mutation of *nusA* or deletion of *nusB*, like mutation of *nusE*, rescued the slow growth of the Δ*suhB* mutant at 37°C (Fig. 1A), indicating that in the absence of SuhB, the “Nus complex” was inhibitory for growth. Since each of the *nus* suppressor alleles also affects translation (22), we tested whether the suppressive effect is simply due to a defect in translation. We constructed derivatives of Δ*suhB* W3110 with additional mutations in each of *infB*, *rpsA*, and *rpsE*. These mutations were originally isolated as suppressors of a growth-defective *secY* mutant (39), and all cause defects in translation. However, none of these mutations reversed the cold sensitivity of the Δ*suhB* mutant (see Fig. S1 in the supplemental material), indicating that the suppressive effect of *nus* mutants is not simply due to a defect in translation.

We next used transcriptome sequencing (RNA-seq) to compare the effect on global RNA levels of deleting *nusB* or *suhB*. For both the *nusB* and *suhB* deletions, more than 25% of all genes had significantly altered RNA levels compared to wild-type cells (>2-fold difference; false discovery rate [FDR], <0.05) (Fig. 1B and C). However, only 3% of all genes were significantly different between the two mutants (Fig. 1D). We concluded that the SuhB function is closely related to that of the Nus factors.

### SuhB is required for BoxA-mediated antitermination in a reporter system.

**FIG 2:**
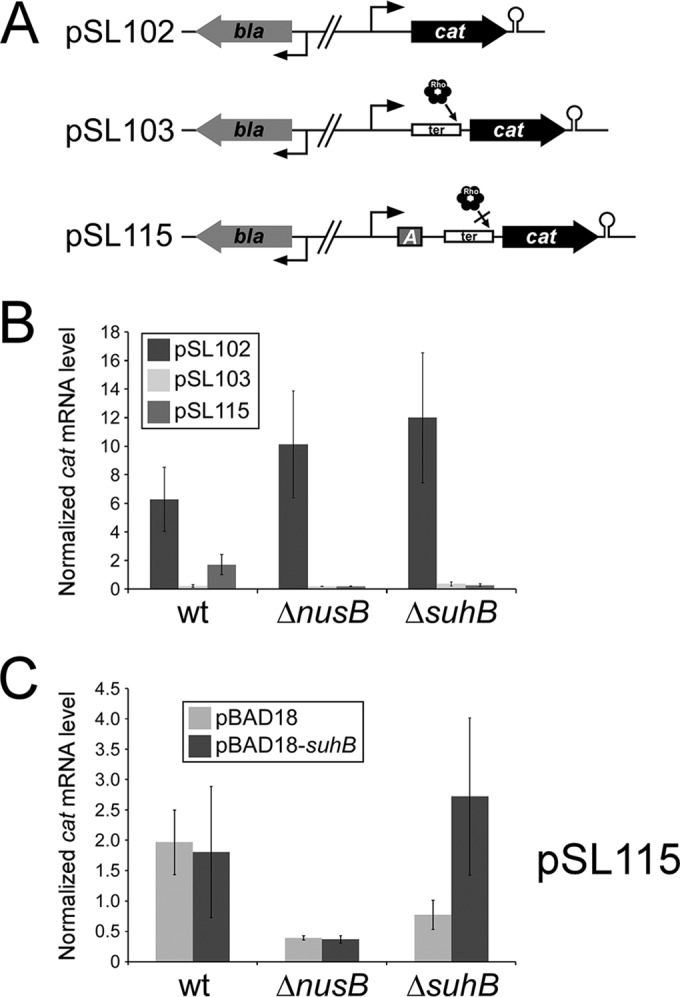
SuhB is required for BoxA-mediated antitermination. (A) Plasmids used for the antitermination reporter assay. pSL102 contains *cat* under control of a constitutive promoter. pSL103 is a derivative of pSL102 that includes a 567-bp noncoding sequence in the 5′-UTR of *cat*. pSL115 is a derivative of pSL103 that includes a BoxA-containing sequence from the rRNA leader in the 5′-UTR. All plasmids contain *bla*, which served as a normalization control. (B) qRT-PCR was used to determine the levels of *cat* mRNA relative to *bla* mRNA for each of the three plasmids in wild-type, Δ*nusB*, and Δ*suhB* cells, as indicated. Data represent an average of three independent, biological replicates. Error bars indicate 1 standard deviation above and below the mean. (C) qRT-PCR was used to determine the levels of *cat* mRNA relative to *bla* mRNA for pSL115 in wild-type, Δ*nusB*, and Δ*suhB* cells that contained either empty vector (pBAD18) or expressed SuhB from a plasmid (pBAD18-*suhB*).

A plasmid-based reporter assay has been previously described for BoxA-mediated antitermination (Fig. 2A) (20, 21). We used this reporter assay to determine whether SuhB is required for antitermination. There are three reporter plasmids, all of which use *cat* as the reporter; levels of *cat* RNA are measured using quantitative reverse transcription-PCR (qRT-PCR) (with results normalized to levels of *bla* RNA, which is expressed constitutively from the same plasmid). In the first of the three plasmids, pSL102, *cat* RNA has a short 5′-untranslated region (UTR) and is constitutively expressed at a high level. The second plasmid, pSL103, is based on pSL102 but has a 567-bp noncoding sequence inserted in the 5′-UTR, leading to premature Rho-dependent termination of the *cat* mRNA. The third plasmid, pSL115, is based on pSL103 but also has a *boxA*-containing sequence from *E. coli* rRNA inserted in the 5′-UTR, preventing Rho-dependent termination (20). Expression of *cat* in wild-type cells was highest for pSL102, greatly reduced for pSL103, and partially rescued by the *boxA* in pSL115 (Fig. 2B), consistent with findings in previous studies (20, 21). As expected, BoxA-mediated antitermination from pSL115 was not observed in Δ*nusB* cells (Fig. 2B). Similarly, we did not observe any BoxA-mediated antitermination in Δ*suhB* cells (Fig. 2B). The defect in antitermination in Δ*suhB* cells could be complemented by overexpression of *suhB* from a plasmid (Fig. 2C). We concluded that SuhB is required for BoxA-mediated antitermination.

### SuhB is not required for N-mediated antitermination in bacteriophage λ.

A previous study (44) indicated that *suhB* (referred to as *ssyA* in that study) is not required for Nus factor function in the λ system. However, this phenotype was not investigated in detail. Hence, we measured plaque formation by a Δ*suhB* mutant with wild-type λ and each of the λ r32 and λ r14 mutants that carry insertion elements enhancing termination and sensitizing the system to *nus* mutations (45). As controls, we measured plaque formation with wild-type *E. coli* cells, and with 2 *nus* mutant strains, Δ*nusB* and *nusA1*. As expected, the *nus* mutant strains were defective in plaque formation by wild-type λ and were even more defective in plaque formation by λ r32 and λ r14 (see Fig. S2 in the supplemental material). In contrast, the Δ*suhB* mutant was not defective in plaque formation by the wild type, λ r32, or λ r14 (see Fig. S2). As an additional control, we measured plaque formation by a λ *nin5* mutant that lacks terminators and is consequently unaffected by *nus* mutations. As expected, this mutant λ formed plaques efficiently with all strains tested (see Fig. S2). We concluded that SuhB is not required for N-mediated antitermination in λ.

### SuhB associates with elongating RNAP at rRNA loci in a NusB-dependent manner.

**FIG 3:**
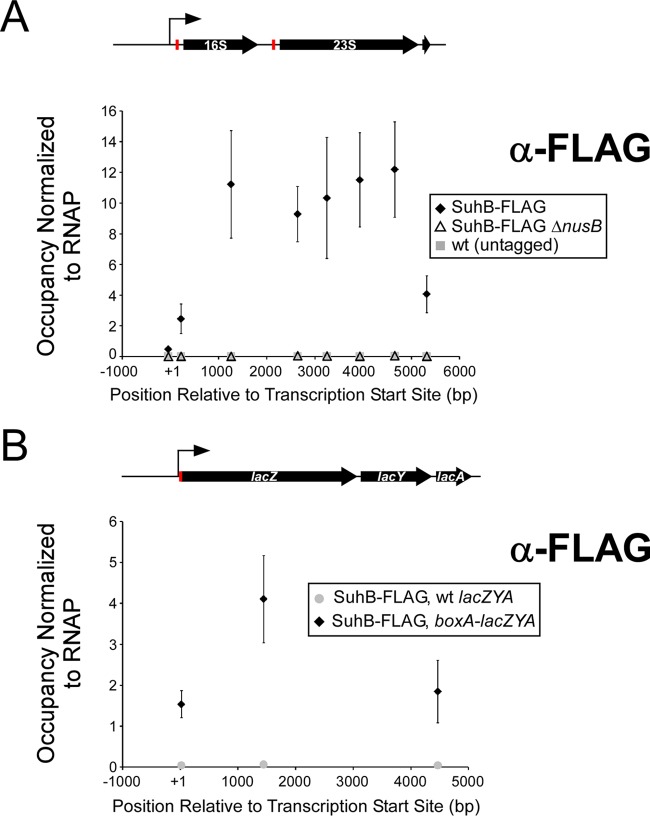
SuhB is recruited to elongating RNAP in a *boxA*-dependent, NusB-dependent manner. (A) The level of SuhB-FLAG_3_ and RNAP (β) association across rRNA loci was measured using ChIP-qPCR for wild-type (wt; SuhB-FLAG) and Δ*nusB* (SuhB-FLAG Δ*nusB*) cells. As a control, the experiment was performed with untagged, wild-type MG1655. Data points indicate the level of ChIP signal using the FLAG antibody (i.e., association of SuhB or the negative control; α-FLAG), normalized to that of RNAP β. The position of each data point on the *x* axis indicates the center of the PCR amplicon. A schematic of an rRNA locus is drawn to scale, aligned with the data. The *boxA* sequence is represented by a red rectangle. (B) The level of SuhB-FLAG_3_ and RNAP (β) association across *lacZYA* was measured using ChIP-qPCR for wild-type cells (SuhB-FLAG wt *lacZYA*) and cells in which a *boxA*-containing sequence had been inserted in the *lacZYA* 5′-UTR (SuhB-FLAG, *boxA*-*lacZYA*). Data points indicate the level of ChIP signal detected using the FLAG antibody (i.e., association of SuhB or the negative control), normalized to that of RNAP β. The position of each data point on the *x* axis indicates the center of the PCR amplicon. Note that we grew cells in the presence of the inducer isopropyl-β-d-thiogalactopyranoside, but *lacZYA* was constitutively transcribed in the *boxA*-*lacZYA* strain because the *boxA* insertion interrupts a binding site for the LacI repressor (65). A schematic of the *lacZYA* operon is drawn to scale, aligned with the data. The inserted *boxA* sequence is represented by a red rectangle in the 5′-UTR. Data represent averages of three independent, biological replicates. Error bars indicate 1 standard deviation above and below the mean.

We next determined the association of SuhB and RNAP (β) with rRNA loci by using chromatin immunoprecipitation (ChIP) coupled with qPCR (ChIP-qPCR). ChIP-qPCR measures association of proteins with DNA. Although NusB, NusE, and any associated proteins are expected to associate with rRNA rather than its DNA, we expected that they would be detectable using ChIP-qPCR due to association with DNA via the elongating RNAP. Indeed, we detected a robust ChIP-qPCR signal for SuhB across the rRNA loci (average of the 7 nearly identical copies, with the exception of the most upstream amplicon, which is specific to *rrnB*), beginning approximately at the position of the *boxA* sequence. Data were normalized to the level of RNAP (β) occupancy (Fig. 3A). Association of SuhB was completely dependent upon the presence of NusB (Fig. 3A). Together, these data indicate that SuhB association with elongating RNAP complexes requires NusB (and presumably NusE) at BoxA sites in rRNA. The dependence of the SuhB association with rRNA loci on the presence of NusB is not due to reduced levels of SuhB in Δ*nusB* cells. In fact, SuhB mRNA levels and SuhB protein levels are increased in a Δ*nusB* mutant relative to wild-type cells (Fig. 1B; see also Fig. S3 in the supplemental material).

### SuhB is recruited to RNAP by BoxA in a nonribosomal context.

Previous studies have shown that a *boxA*-containing sequence from rRNA loci is functional when inserted upstream of a heterologous gene on a plasmid (29, 30). We introduced a 32-nucleotide (nt) BoxA-containing sequence from the rRNA leader into the 5′-UTR of *lacZYA*. We measured the association of SuhB at three positions across the *lacZYA* operon by using ChIP-qPCR. We detected robust ChIP-qPCR signal for SuhB across *lacZYA* (Fig. 3B). In contrast, we detected very little association of SuhB across *lacZYA* in a strain with a wild-type *lac* operon (i.e., no *boxA*) (Fig. 3B).

### Robust rRNA antitermination occurs in the absence of Nus factors or SuhB.

**FIG 4:**
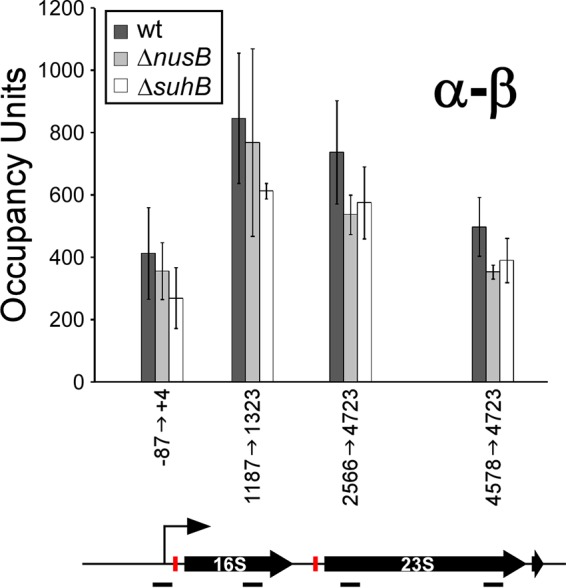
Limited role for NusB and SuhB in rRNA antitermination. ChIP-qPCR was used to measure the association of RNAP (β) at positions across the inserted phage rDNA in wild-type cell, Δ*nusB* cells, and Δ*suhB* cells. Values indicate the background-subtracted fold enrichment above a control region in the transcriptionally silent *bglB* gene (see Materials and Methods). A schematic of the rRNA loci is drawn to scale and aligned with the data. Horizontal black lines indicate the position of the amplicons used for PCR quantification of the ChIP signal. *boxA* sequences are represented by red boxes. Data represent averages of three independent, biological replicates. Error bars indicate 1 standard deviation above and below the mean.

The described function of Nus factors is to prevent Rho-dependent termination of transcription within rRNA. Hence, deletion of *nusB* or *suhB* would be expected to result in reduced levels of RNAP occupancy throughout rRNA loci, except at the very 5′ ends. We used ChIP-qPCR to measure association of RNAP (β subunit) across the rRNA loci in wild-type cells, the Δ*nusB* mutant, and the Δ*suhB* mutant. We detected only a slight reduction (~25%) in the level of RNAP association at any point across the rRNA loci between the wild-type and mutant strains (Fig. 4). Moreover, we detected a similar reduction in the level of RNAP association in the rRNA promoter region, well upstream of the leader *boxA*. Thus, our data suggest that Nus factors and SuhB are required for maximal transcription initiation and that rRNA is resistant to Rho-dependent termination, even in the absence of Nus factors.

### SuhB is likely required for proper ribosome biogenesis.

A recent study showed that, like the Δ*suhB* mutant strain, the cold sensitivity of a Δ*nusB* strain is rescued by an *rnc* mutation (22). Moreover, deletion of *nusB* causes a translation defect that rescues the temperature sensitivity of a *secY* mutation (39, 41). This rescue is reversed if *rnc* is deleted (22). To investigate a possible role of SuhB in rRNA maturation, we determined the genetic interactions of *suhB* with *rnc* and *secY*. We first confirmed that deletion of *rnc* prevents cold sensitivity in W3110 Δ*suhB* (see Fig. S4 in the supplemental material), consistent with our suppressor screen (see Table S3 in the supplemental material) and a previous study (40). We also confirmed that deletion of *suhB* rescues the temperature sensitivity of a *secYts24* mutant, consistent with a previous study (36), and we observed that rescue of *secYts24* temperature sensitivity by the Δ*suhB* mutant, as with the Δ*nusB* mutant, was reversed when *rnc* was deleted. Thus, the genetic interactions of *suhB* mirror those of *nusB*, supporting a role for SuhB in rRNA folding and ribosome biogenesis. In this regard, mutations of *nusB* and *suhB* have been shown to affect ribosome function by reducing translation elongation rates (36, 39).

### SuhB is an rRNA chaperone that promotes loop formation between BoxA and RNAP.

**FIG 5:**
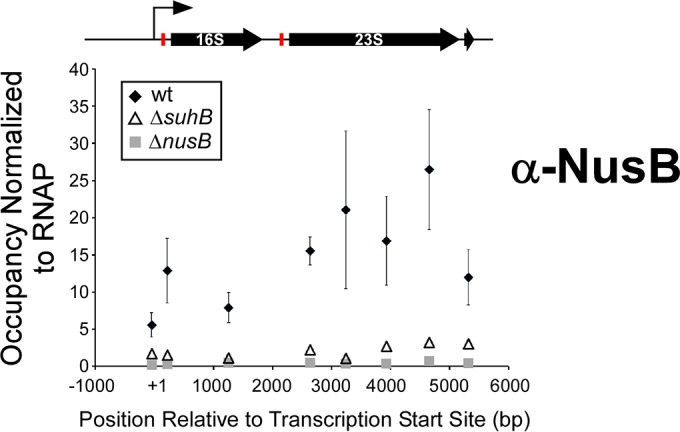
NusB association with elongating RNAP at rRNA loci is dependent upon SuhB. The level of NusB and RNAP (β) association across rRNA loci was measured using ChIP-qPCR for wild-type and Δ*suhB* and Δ*nusB* strain cells. Data points indicate the level of NusB ChIP signal normalized to that of RNAP β. The position of each data point on the *x* axis indicates the center of the PCR amplicon. A schematic of the rRNA loci is drawn to scale, aligned with the data. *boxA* sequences are represented by red boxes. Data represent averages of three independent, biological replicates. Error bars indicate 1 standard deviation above and below the mean.

Our data are consistent with a role for SuhB in rRNA maturation, as has been previously proposed for other Nus factors (22). We hypothesized that SuhB is required for Nus factor-mediated loop formation in rRNA by interacting simultaneously with elongating RNAP complexes and NusB/E-bound BoxA. Consistent with this model, our data strongly suggest a NusB-dependent association of SuhB with transcription elongation complexes (we detected an association of SuhB across transcription units) (Fig. 3). Moreover, SuhB has been previously reported to bind RNAP *in vitro*, albeit weakly, and a recent study reported an association of SuhB with RNAP *in vivo* in *Pseudomonas aeruginosa* (46). To test the model, we used ChIP-qPCR to measure the association of NusB across rRNA loci in the wild-type and Δ*suhB* mutant strains of MG1655. We detected a robust association of NusB across rRNA loci, downstream of the first *boxA*, in wild-type cells (Fig. 5). Consistent with the model, NusB association with rRNA loci was almost completely dependent upon SuhB (Fig. 5).

## DISCUSSION

### SuhB is a new player in rRNA regulation.

The role of Nus factors in regulation of rRNA has been studied extensively. However, failure to fully reconstitute antitermination *in vitro* with purified Nus factors suggested that at least one component of the machinery was missing (27, 42). We have identified SuhB as such a factor. SuhB is recruited by BoxA and Nus factors, and remains associated with RNAP throughout transcription of rRNA (Fig. 3A). Much of our understanding of Nus factor function comes from work on antitermination in λ phage, and importantly in this regard, a *suhB* mutant does not have a Nus mutant phenotype in λ (44) (see Fig. S2 in the supplemental material), perhaps explaining why the rRNA function of SuhB has remained undiscovered for so long. This work also serves to highlight that the function of Nus proteins in λ antitermination is fundamentally different from their main function in regulating rRNA synthesis and folding.

SuhB has a well-characterized enzymatic function as an IMPase (33). Thus, SuhB appears to serve two unrelated functions. It is possible that its function in rRNA regulation is connected to its IMPase activity; however, at least in *E. coli*, there is no obvious metabolic requirement for IMPase activity (34). SuhB is widely conserved, and mutants of *suhB* and its homologues have been shown to be inviable or slow-growing in several species (33, 47–50). Moreover, mutation of *suhB* in *Burkholderia cenocepacia* and *P. aeruginosa* affects expression of large numbers of genes, including genes required for virulence (51, 52); in *P. aeruginosa*, the resulting phenotype has been linked to defective ribosome function (46). Hence, it is likely that the function of SuhB in regulating rRNA is phylogenetically widespread.

### The major function of Nus factors and SuhB at rRNA is not antitermination.

Almost all studies of Nus factors and their role in rRNA regulation have been predicated on the idea that the function of Nus factors is to prevent Rho-dependent termination within rRNA (53). Our data show that RNAP occupancy across rRNA is only slightly reduced following deletion of *nusB* or *suhB*, and that this decrease is likely due to reduced transcription initiation (Fig. 4), although we cannot rule out changes in the RNAP ChIP signal being masked by an altered RNAP conformation or altered transcription elongation rates in the mutant strains. We therefore propose that most transcription of rRNA is inherently resistant to Rho, perhaps due to the high degree of secondary structure and the association of many proteins with the RNA, features known to prevent Rho function (54). Two lines of evidence support our observation that the function of Nus factors at rRNA is not antitermination. First, although early studies reported an increase in 16S relative to 23S rRNA in strains with mutant or titrated Nus factors (23, 24), the probe used to detect 16S rRNA was found to hybridize to a sequence beginning >1,000 nt downstream of BoxA. This is inconsistent with antitermination by Nus factors, since Rho would be expected to terminate transcription much further upstream (55). Second, the increase in 16S relative to 23S rRNA in strains with mutant Nus factors can be reversed by mutation of *rnc* (encodes RNase III) (22). This is also inconsistent with antitermination by Nus factors, since RNase III is not known to play a role in Rho termination.

Although our data support a role for Nus factors and SuhB at rRNA unconnected to antitermination, two observations suggest that Nus factors may prevent Rho-dependent termination of transcription in other contexts. First, NusB and BoxA were required for antitermination *in vitro* when a template containing the *trpt*′ sequence that induces Rho-dependent termination was used (27). Second, *in vivo* expression of a reporter gene is repressed following insertion of a long, noncoding sequence in the 5′-UTR (Fig. 2) (19). This repression is dependent upon Rho (19) and is prevented by inclusion of an upstream BoxA sequence from rRNA (20), in a NusB-dependent, NusG-dependent manner (21).

### Mechanism of Nus/SuhB-mediated rRNA maturation.

Our data are consistent with a role for SuhB in 30S ribosome biogenesis, as has been previously described for Nus factors (22). Given the limited role for Nus factors and SuhB in rRNA antitermination, we propose that control of rRNA maturation is the primary function of these proteins in regulation of ribosome biogenesis. Based on the defects in ribosome biogenesis associated with *nus* mutants, the suppression of these defects by mutation of *rnc* (encodes RNase III), and the proximity of the rRNA leader BoxA to the upstream arm of the 16S stem-loop, a model has been proposed (22) in which Nus factors are required for tethering of BoxA RNA to elongating RNAP. Thus, Nus factors promote proper cotranscriptional folding of rRNA. Our data support and extend this model, implicating SuhB in facilitating loop formation. Deletion of *suhB* abolishes the NusB ChIP signal across rRNA loci (Fig. 5). The NusB ChIP signal at rRNA loci is presumably due to association of NusB with the elongating RNAP. Consistent with this, the NusB ChIP signal is observed across all regions of rRNA loci downstream of the first BoxA site. Therefore, loss of NusB ChIP signal at rRNA loci in Δ*suhB* cells indicates that NusB no longer associates with elongating RNAP. This could be due to loss of binding to BoxA RNA. However, NusB and NusE bind with high affinity to the BoxA RNA in the absence of any other proteins *in vitro* (56), making it highly unlikely that SuhB is required for association of NusB with rRNA *in vivo*. We conclude that SuhB is required for loop formation between NusB/E-bound BoxA and the elongating RNAP complex. This is consistent with the known *in vivo* interaction between SuhB and RNAP in *P. aeruginosa* (46), although the weakness of the interaction between *E. coli* SuhB and RNAP *in vitro* suggests that other proteins may contribute to the association of SuhB with elongating RNAP (35).

### Conclusions.

In summary, we have redefined the role of the Nus “antitermination” proteins at rRNA, and we have identified SuhB as a novel member of this complex. Our data indicate that while these proteins are able to prevent Rho-dependent termination in the plasmid context (Fig. 2), their major function in ribosome biogenesis is to promote correct ribosome assembly, and this occurs due to Nus factor-mediated loop formation in the nascent rRNA. Loop formation is mediated by SuhB, which likely bridges the gap between the elongating RNAP (bound to NusA and NusG), and the NusB/E-bound BoxA. Important questions remain. The architecture of the complex is still unclear, as is the role of NusA and NusG. Moreover, how these proteins prevent Rho-dependent termination (in the appropriate context) and increase the transcription elongation rate are yet to be determined. Lastly, we cannot rule out the possibility that other members of the complex are yet to be identified.

## MATERIALS AND METHODS

### Strains and plasmids.

All strains and plasmids are listed in Table S1 in the supplemental material. Oligonucleotides used for strain construction, plasmid construction, and PCRs are listed in Table S2 in the supplemental material. Strains MG1655 Δ*thyA* Δ*suhB*::*thyA* (VS070) and MG1655 Δ*thyA* Δ*nusB*::*thyA* (JW022) were generated using FRUIT (57). The *thyA*-containing PCR products were amplified using oligonucleotides JW4154/JW4155 and JW3611/JW3612 for Δ*suhB*::*thyA* and Δ*nusB*::*thyA*, respectively. SuhB was C-terminally epitope tagged in MG1655 with three FLAG epitopes by using FRUIT, to give VS066 (57). The *thyA*-containing PCR product for tagging was amplified using oligonucleotides JW3246/JW3247. The MG1655 Δ*thyA* Δ*suhB*-FLAG_3_ Δ*nusB*::*thyA* strain (JW024) was generated by replacing *nusB* with *thyA* rather than replacing *thyA* at its native locus (57). FRUIT (57) was used to insert a *boxA*-containing sequence upstream of *lacZ* in VS066 to give VS077. The *thyA*-containing PCR product was amplified using oligonucleotides JW5068/JW5069. *thyA* was replaced using a PCR product that contained *boxA*-containing sequence from rRNA, generated using oligonucleotides JW5014, JW5015, JW5017, and JW5180. *rfaH* was disrupted by precisely replacing the open reading frame (ORF) with a chloramphenicol resistance cassette in DY330, a recombinogenic derivative of W3110, as described elsewhere (58), to give NB359. The *mfd* ORF was similarly replaced with a kanamycin resistance cassette to give NB365. Δ*nusB*::*cat* (NB421) (58), Δ*rnc*::*cat* (NB97) (59), and Δ*suhB*::*kan* (NB760) (35) derivatives of W3110 have been described previously. The Δ*suhB*::*bla* knockout (NB762) was constructed in the same manner as Δ*suhB*::*kan* (35). The Δ*suhB*::*bla* mutation was P1 transduced into wild-type W3110 cells to make NB762. Other mutations were P1 transduced into NB762. *secYts24* Δ*suhB* Δ*rnc* double and triple mutants were constructed based on a *secYts*24 strain (IQ85) provided by K. Ito that contained a Tet^r^ determinant Tn*10*. These cells were rendered tetracycline sensitive by using the chlortetracycline method (60), yielding strain NB50. The Δ*suhB* and Δ*rnc* mutations were then P1 transduced into NB50 to obtain the respective double mutants NB76 and NB53. The Δ*rnc*::*cat secYts24* Δ*suhB* triple mutant (NB80) was constructed by P1 transducing Δ*rnc*::*cat* into the double *secYts24* Δ*suhB* mutant.

The *ssy* mutations described here were isolated in MC4100 (39). The *suhB ssy* double mutants were made by P1 transduction of the *suhB*<>*bla* or *suhB*<>*kan* knockouts into the *ssy*-carrying strains by selection for the appropriate drug marker.

To construct pBAD18-*suhB* (pDMA027), the *suhB* ORF and the Shine-Dalgarno sequence from pBAD24 (61) were PCR amplified using oligonucleotides JW4362/JW4363 and cloned into the NheI restriction site of pBAD18-Kan (61) using an In-Fusion kit (Clontech).

### Generation of *rho15*::*bla.*

The *rho15* mutant has been described previously (62), but we removed all but the first 21 bp of the IS*1* element and replaced it with *bla* to give NB966. Unlike the original *rho15* mutant that has an intact IS*1* (62), the mutant we constructed was not temperature sensitive at 42°C, suggesting that the temperature sensitivity of the original mutant is due to the IS*1* element rather than mutation of *rho*.

### SuhB suppressor screen.

Five cultures of MG1655 Δ*thyA* Δ*suhB*::*thyA* were grown overnight from single colonies at 37°C in LB. Five microliters of each overnight culture was spread on LB agar and incubated at 30°C, the nonpermissive temperature for Δ*suhB* mutants. One suppressor mutant colony was selected from each plate. *rnc* was PCR amplified from colonies using oligonucleotides JW836-JW837, and the PCR products were sequenced to identify the presence, if any, of suppressor mutations. Genomic DNA from a strain with wild-type *rnc* was prepared using a DNeasy blood and tissue kit (Qiagen). A DNA library was prepared using a Nextera kit (Illumina). The library was sequenced (paired-end reads) using an Illumina MiSeq instrument. Sequence reads were aligned to the reference *E. coli* MG1655 genome for single nucleotide polymorphism and structural variant detection using the CLC genomic workbench (with default parameters). Mutations listed as “homozygous” in the output file were presumed to be genuine.

### Determining whether a *nusE* mutation is necessary to suppress the cold sensitivity of a Δ*suhB* strain.

*E. coli* strain CAG12071 contains a *smg-3082*::Tn*10* insertion that is predicted to cotransduce with *nusE* ~44% of the time. We used P1 transduction (63) with tetracycline selection to transfer the *tetA* gene from Tn*10* into the MG1655 Δ*thyA* Δ*suhB*::*thyA* derivative that contains a *nusE* mutation (VS093) and that is no longer cold sensitive. We initially selected for tetracycline-resistant transductants by plating on tetracycline-containing LB agar at 42°C. We then patched colonies on plates grown at 30°C and counted the number of surviving strains. As a control, we patched colonies on plates grown at 42°C. We then selected 10 strains that were cold sensitive and 10 that were not, PCR-amplified *nusE*, and sequenced using conventional Sanger sequencing.

### λ spot titers.

Single colonies from LB agar were inoculated into 5 ml of LB broth at 42°C for overnight cultures. Two hundred microliters of each overnight culture was added to 2.5 ml of molten tryptone broth (TB) top agar, which was overlaid on TB agar plates. Twenty microliters each of serial dilutions (10^−2^, 10^−4^, 10^−6^, and 10^−7^) of λ phage lysates was spotted on the bacterial lawns and incubated at 37°C overnight.

### ChIP-qPCR.

All cultures for ChIP-qPCR were grown in LB at 37°C to mid-exponential phase. ChIP-qPCR was performed as described previously (64), using anti-FLAG mouse monoclonal (Sigma), anti-RpoB mouse monoclonal (NeoClone), or anti-NusB rabbit polyclonal (gift from Evgeny Nudler) antibody. Occupancy units are a measure of binding and were calculated by first determining the level of enrichment relative to a nontranscribed control region (within the *bglB* gene) and then subtracting 1 (i.e., subtracting the background). Background subtraction allows for a more quantitative comparison of values between experiments and between target regions. Consider a case where one region is not enriched relative to the control (1-fold enrichment), and another region is 2-fold enriched. The ratio of enrichment is 2, which does not reflect the fact that one region is not enriched at all. Amplicons were generated using oligonucleotide pairs JW125/JW126 (*bglB* ORF), JW4861/JW4862 (rRNA), JW2747/JW2748 (rRNA), JW4865/JW4866 (rRNA), JW4869/JW4870 (rRNA), JW4871/JW4872 (rRNA), JW4873/JW4874 (rRNA), JW4875/JW4876 (rRNA), JW4877/JW4878 (rRNA), JW166/JW167 (*lacZ* upstream region), JW186/JW187 (within *lacZ*), and JW123/JW124 (within *lacY*/*lacA*).

### RNA-seq.

Two independent biological replicates of MG1655, MG1655 Δ*thyA* Δ*suhB*::*thyA* and MG1655 Δ*thyA* Δ*nusB*::*thyA*, were each grown in LB to mid-exponential phase. RNA-seq and associated data analysis were performed as described previously (64).

### qRT-PCR.

Strain MG1655, MG1655 Δ*thyA* Δ*suhB*::*thyA*, or MG1655 Δ*thyA* Δ*nusB*::*thyA* was transformed with plasmid pSL102, pSL103, or pSL115 (20). Cells were grown in LB supplemented with ampicillin at 37°C to mid-exponential phase. For SuhB complementation, pBAD18 vector and pBAD18-*suhB* (pDMA027) were also transformed into cells, and cells were grown in LB supplemented with kanamycin and ampicillin to an optical density at 600 nm of 0.4 before induction with arabinose (0.2%, final concentration) for 30 min. Aliquots of 1.5 ml of cell culture were centrifuged, and pellets were resuspended in 1 ml RNAzol RT (Molecular Research Center Inc.). RNA was prepared according to the manufacturer’s instructions. RNA was DNase treated (TURBO DNase I; Life Technologies) and reverse transcribed using SuperScript III reverse transcriptase (Invitrogen). Quantitative real-time PCR on biological triplicates was performed using the ABI 7500 PCR machine. Amplicons were generated using oligonucleotide pairs JW4337/JW4338 (*bla*) and JW4333/JW4334 (*cat*).

### Western blotting.

Cell extracts were separated on a gradient polyacrylamide gel and transferred to a PVDF-Plus membrane (GE Healthcare) by electrophoresis. The membrane was probed with a 1:4,000 dilution of anti-FLAG mouse monoclonal M2 antibody (Sigma-Aldrich) or a 1:4,000 dilution of control anti-RpoC antibody (NeoClone). Blots were then probed with a 1:10,000 dilution of secondary goat anti-mouse horseradish peroxidase-conjugated antibody. Blots were developed using the Immun-Star Western kit (BioRad) or the SuperSignal Femto kit (Pierce).

### Nucleotide sequence accession number.

Raw sequencing data from the suppressor screen and RNA-seq analyses have been deposited with the EBI ArrayExpress database and assigned accession number E-MTAB-4240.

## SUPPLEMENTAL MATERIAL

Figure S1  Defective translation is insufficient to suppress the cold-sensitive phenotype of a *suhB* mutant. Growth phenotypes on LB agar at 30°C, 37°C, and 42°C, of wild-type MC4100 and *ssyG40* (*infB* that causes a translation defect), *ssyF29* (*rpsA* mutation that causes a translation defect), *ssyE36* (*rpsE* mutation that causes a translation defect), Δ*suhB*, Δ*suhB ssyG40*, Δ*suhB ssyF29*, and Δ*suhB ssyE36* mutant strains. Download Figure S1, PDF file, 1.3 MB

Figure S2  SuhB is not required for N-mediated antitermination in λ. Plaque assays for wild-type λ, λ *nin5*, λ *r32*, and λ (*r14*) on lawns of wild-type W3110 and *nusA1*, Δ*nusB*, and Δ*suhB* derivatives. Dilutions of λ are indicated below each plate. Download Figure S2, PDF file, 2.1 MB

Figure S3  Deletion of *nusB* results in an increase in SuhB protein levels. A representative Western blot using antibody raised against the RNAP β′ subunit (RpoC) or the FLAG epitope, for wild-type untagged cells (first lane), *nusB*^+^ cells expressing SuhB-FLAG_3_ (second lane), and Δ*nusB* cells expressing SuhB-FLAG_3_ (third lane). Download Figure S3, PDF file, 0.1 MB

Figure S4  Deletion of *rnc* suppresses the cold-sensitive phenotype of a *suhB* mutant strain. Growth phenotypes on LB agar of the wild-type W3110, Δ*rnc*, Δ*suhB*, and Δ*rnc* Δ*suhB* strains. Download Figure S4, PDF file, 0.2 MB

Table S1  Bacterial strains, bacteriophage, and plasmids used in this study. Table S1, PDF file, 0.2 MB

Table S2  Oligonucleotides used in this study. Table S2, PDF file, 0.1 MB

Table S3  Mutants that suppress the cold sensitivity of a Δ*suhB* strain. Table S3, PDF file, 0.2 MB
