# Supplementary figures and images for: SuhB Associates with Nus Factors To Facilitate 30S Ribosome Biogenesis in Escherichia coli
Source: mBio. 2016 Mar 15;7(2):e00114-16. doi: 10.1128/mBio.00114-16 (PMC4807359; doi:10.1128/mBio.00114-16)

Figure S1

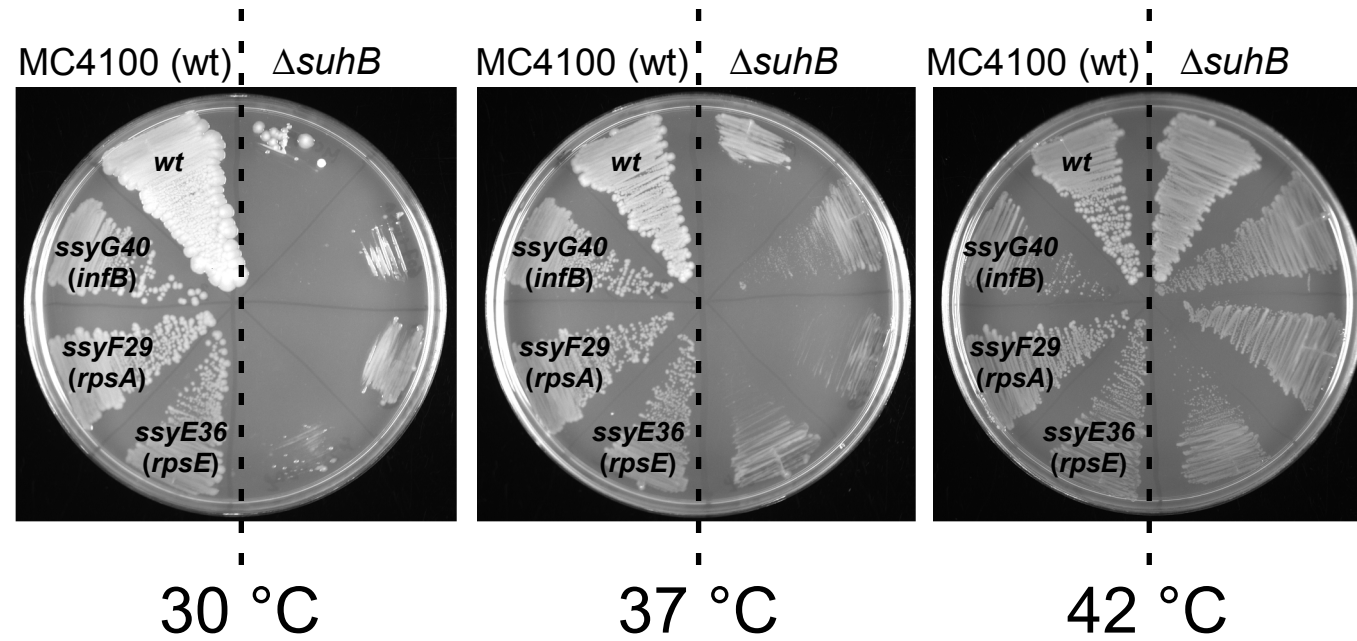

Supplement: Figure S1 — Defective translation is insufficient to suppress the cold-sensitive phenotype of a suhB mutant. Growth phenotypes on LB agar at 30°C, 37°C, and 42°C, of wild-type MC4100 and ssyG40 (infB that causes a translation defect), ssyF29 (rpsA mutation that causes a translation defect), ssyE36 (rpsE mutation that causes a translation defect), ΔsuhB, ΔsuhB ssyG40, ΔsuhB ssyF29, and ΔsuhB ssyE36 mutant strains. Download [file mbo002162727sf1.pdf]

Figure S2

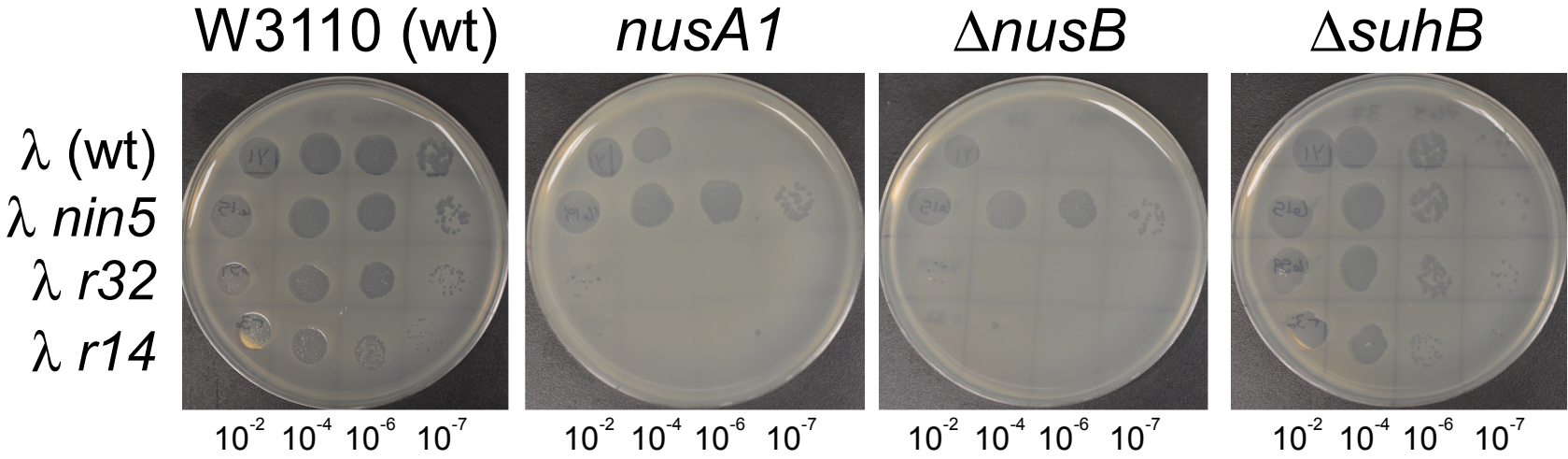

Supplement: Figure S2 — SuhB is not required for N-mediated antitermination in λ. Plaque assays for wild-type λ, λ nin5, λ r32, and λ (r14) on lawns of wild-type W3110 and nusA1, ΔnusB, and ΔsuhB derivatives. Dilutions of λ are indicated below each plate. Download [file mbo002162727sf2.pdf]

Figure S3

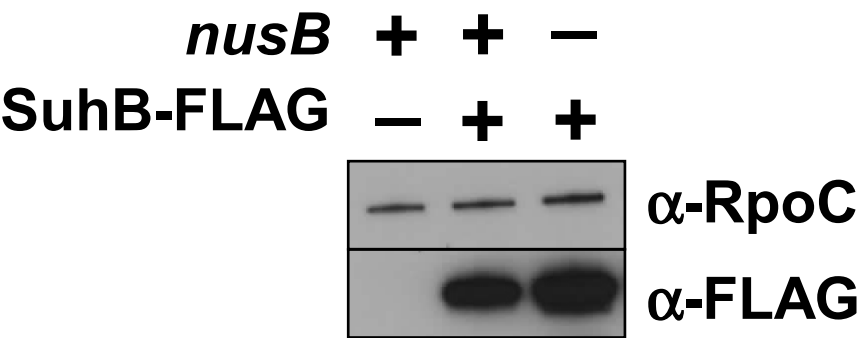

Supplement: Figure S3 — Deletion of nusB results in an increase in SuhB protein levels. A representative Western blot using antibody raised against the RNAP β′ subunit (RpoC) or the FLAG epitope, for wild-type untagged cells (first lane), nusB+ cells expressing SuhB-FLAG3 (second lane), and ΔnusB cells expressing SuhB-FLAG3 (third lane). Download [file mbo002162727sf3.pdf]

Figure S4

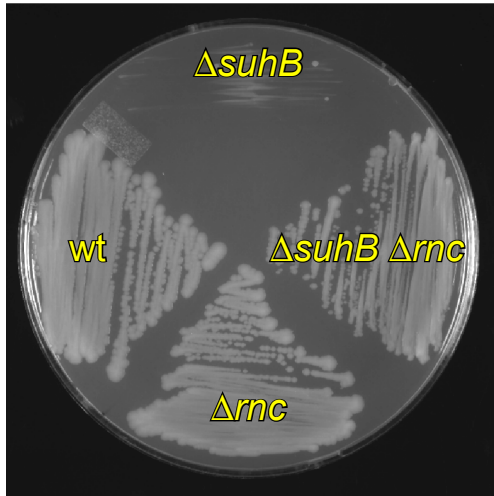

Supplement: Figure S4 — Deletion of rnc suppresses the cold-sensitive phenotype of a suhB mutant strain. Growth phenotypes on LB agar of the wild-type W3110, Δrnc, ΔsuhB, and Δrnc ΔsuhB strains. Download [file mbo002162727sf4.pdf]
