# Supplementary material for: SuhB Associates with Nus Factors To Facilitate 30S Ribosome Biogenesis in Escherichia coli
Source: mBio. 2016 Mar 15;7(2):e00114-16. doi: 10.1128/mBio.00114-16 (PMC4807359; doi:10.1128/mBio.00114-16)
Supplement: Table S1 — Bacterial strains, bacteriophage, and plasmids used in this study. [file mbo002162727st1.pdf]

Table S1. Bacterial strains, bacteriophage, and plasmids used in this study

***E. coli* Strains**		
MG1655	F^- λ^- Δ*ilvG rfb-50 rph-1*	(1)
W3110	F^- λ^- *rph-1* INV(*rrnD*, *rrnE*)	(2)
MC4100	F- [araD139]B/r Δ(*argF-lac*)169 λ^- $e14^-$ *flhD5301* Δ(*fruK-yeiR*)725 (fruA25) *relA1 rpsL150 rbsR22* Δ(*fimB-fimE*)632(::IS1) *deoC1*	(3)
DY330	W3110 Δ*lacU169 gal490* λ*cI857* Δ(*cro-bioA*)	(4)
NB760	W3110 Δ*suhB*::*kan*	(5)
NB762	W3110 Δ*suhB*::*bla*	This study
NB97	W3110 Δ*rnc*::*cat*	(4)
IQ85	*secY24 zhe-33*::Tn*10 thiA* Δ*lacU169 araD rpsL rpsE relA*	(6)
NB50	IQ85 tet^S	This study
NB53	NB50 Δ*rnc*::*cat*	This study
NB76	NB50 Δ*suhB*::*kan*	This study
NB80	NB50 Δ*rnc*::*cat* Δ*suhB*::*kan*	This study
NB23	W3110 *nusAcs10*	(7)
NB966	W3110 *rho15::bla*	This study
NB383	W3110 *rho15::bla* Δ*suhB*::*kan*	This study
NB779	W3110 *nusAcs10* Δ*suhB*::*bla*	This study
NB421	W3110 Δ*nusB*::*cat*	(8)
VS070	MG1655 Δ*thyA* Δ*suhB*::*thyA*	This study
VS088	MG1655 Δ*thyA* Δ*suhB*::*thyA rnc*(Δ225)	This study
VS089	MG1655 Δ*thyA* Δ*suhB*::*thyA rnc*(G634T)	This study
VS091	MG1655 Δ*thyA* Δ*suhB*::*thyA rnc*(C577T)	This study
VS092	MG1655 Δ*thyA* Δ*suhB*::*thyA rnc*(A insertion 554)	This study
VS093	MG1655 Δ*thyA* Δ*suhB*::*thyA nusE*(T50A)	This study
JW022	MG1655 Δ*thyA* Δ*nusB*::*thyA*	This study
NB359	W3110 Δ*rfaH*::*cat*	This study
NB365	W3110 Δ*mfd*::*kan*	This study
NB380	W3110 Δ*suhB*::*bla* Δ*rfaH*::*cat*	This study
NB382	W3110 Δ*suhB*::*bla* Δ*mfd*::*kan*	This study
VS077	MG1655 *boxA-lacZ*	This study
VS066	MG1655 *suhB*-$FLAG_3$	This study
JW024	VS066 Δ*thyA* Δ*nusB*::*thyA*	This study
NB4	MC4100 *ssyG40*	(9)
NB3	MC4100 *ssyF29*	(9)
NB83	MC4100 *ssyE36*	(9)
	MC4100 Δ*suhB*::*kan*	This study
	MC4100 Δ*suhB*::*kan ssyG40*	This study
	MC4100 Δ*suhB*::*kan ssyF29*	This study
	MC4100 Δ*suhB*::*kan ssyE36*	This study
CAG12071	MG1655 *smg3082*::Tn10	(10)
**Bacteriophage**		
λ		NIH Collection
λ *nin5*		NIH Collection
λ *r32*		(11)
λ *r14*		(11)

**Plasmids**		
pET11B	pET11B (empty vector)	Novagen
pET11B-*suhB*	pET11B containing *suhB*	(5)
pBAD18-*kan*	pBAD18-*kan* (empty vector)	(12)
pDMA027	pBAD18-*kan* containing *suhB*	This study
pSL102	*cat* reporter plasmid with no terminator	(13)
pSL103	*cat* reporter plasmid with Rho-dependent terminator	(13)
pSL115	*cat* reporter plasmid with Rho-dependent terminator and *boxA*-containing sequence in the 5' UTR	(13)

References

1. **Blattner FR, Plunkett G, III, Bloch CA, Perna NT, Burland V, Riley M, Collado-Vides J, Glasner JD, Rode CK, Mayhew GF, Gregor J, Davis NW, Kirkpatrick HA, Goeden MA, Rose DJ, Mau B, Shao Y.** 1997. The complete genome of *Escherichia coli* K-12. Science **277:**1453-1474.
2. **Bachmann BJ.** 1972. Pedigrees of some mutant strains of *Escherichia coli* K-12. Bacteriol. Rev. **36:**525-557.
3. **Peters JE, Thate TE, Craig NL.** 2003. Definition of the *Escherichia coli* MC4100 genome by use of a DNA array. J. Bacteriol. **185:**2017-2021.
4. **Yu D, Ellis HM, Lee E-C, Jenkins NA, Copeland NG, Court DL.** 2000. An efficient recombination system for chromosome engineering in *Escherichia coli*. Proc. Natl. Acad. Sci. U.S.A. **97:**5978-5983.
5. **Wang Y, Stieglitz KA, Bubunenko M, Court DL, Stec B, Roberts MF.** 2007. The structure of the R184A mutant of the inositol monophosphatase encoded by suhB and implications for its functional interactions in *Escherichia coli*. J. Biol. Chem. **282:**26989-26996.
6. **Shiba K, Ito K, Yura T, Cerretti DP.** 1984. A defined mutation in the protein export gene within the *spc* ribosomal protein operon of *Escherichia coli*: isolation and characterization of a new temperature-sensitive *secY* mutant. EMBO J. **3:**631-635.
7. **Bubunenko M, Court DL, Al Refaii A, Saxena S, Korepanov A, Friedman DI, Gottesman ME, Alix JH.** 2012. Nus transcription elongation factors and RNase III modulate small ribosome subunit biogenesis in *Escherichia coli*. Mol. Microbiol. **87:**382-393.
8. **Bubunenko M, Baker T, Court DL.** 2007. Essentiality of ribosomal and transcription antitermination proteins analyzed by systematic gene replacement in *Escherichia coli*. J. Bacteriol. **189:**2844-2853.
9. **Shiba K, Ito K, Yura T.** 1986. Suppressors of the *secY24* mutation: identification and characterization of additional ssy genes in *Escherichia coli*. J. Bacteriol. **166:**849-856.
10. **Singer M, Baker TA, Schnitzler G, Deischel SM, Goel M, Dove W, Jaacks KJ, Grossman AD, Erickson JW, Gross CA.** 1989. A collection of strains containing genetically linked alternating antibiotic resistance elements for genetic mapping of *Escherichia coli*. Microbiol. Rev. **53:**1-24.
11. **Tomich PK, Friedman DI.** 1977. Isolation of mutations in insertion sequences that relieve IS-induced polarity, p. 99-107, DNA Insertion Elements, Plasmids and Episomes. Cold Spring Harbor Laboratory Cold Spring Harbor, New York.
12. **Guzman L-M, Belin D, Carson MJ, Beckwith JR.** 1995. Tight regulation, modulation, and high-level expression by vectors containing the arabinose P_{BAD} promoter. J. Bacteriol. **177:**4121-4130.
13. **Li SC, Squires CL, Squires C.** 1984. Antitermination of *E. coli* rRNA transcription is caused by a control region segment containing lambda nut-like sequences. Cell **38:**851-860.
