# Supplementary material for: SuhB Associates with Nus Factors To Facilitate 30S Ribosome Biogenesis in Escherichia coli
Source: mBio. 2016 Mar 15;7(2):e00114-16. doi: 10.1128/mBio.00114-16 (PMC4807359; doi:10.1128/mBio.00114-16)
Supplement: Table S2 — Oligonucleotides used in this study. [file mbo002162727st2.pdf]

Table S2. Oligonucleotides used in this study

Oligonucleotide	Sequence
JW123	GCCTTATCCGACCAACAT
JW124	TTCGCACATATCGGTAAA
JW125	AAGCGAAAATCGGCAATA
JW126	CATGGCCTGCAACATATC
JW166	GGCTTTACACTTTATGCTTCC
JW167	GGTTTTCCCAGTCACGA
JW186	CTGGGGAATGAATCAGG
JW187	CGTACATCGGGCAAATAA
JW836	CGGTAAACTGAAACTGCAG
JW837	GGAAGTGATGGAGATTTTC
JW2747	GGCACTGCTCTTTAACAATTT
JW2748	AAAGTTTGACGCTCAAAGAA
JW3246	ATGCTGGCGAACATGCGTGACGAGTTAAGCGACGCTCTGAAGCGTGGCGGTGGC GACTAC
JW3247	GCGAGGGCGGGTGAGTGATATCACCCGCCTGAGTCATTAGTTGGGAGCTCACTAC TTGTC
JW3611	GTATTGAAAGCCATCAAGGCCTGAAATTAGTAAGGGGAAATCCTAGACAGCTGC ATGCAT
JW3612	CGGCGATCATGGAACGGTCTTCCGTGAATCTACCGGCCTGGATAGTGTAGGCTGG AGCTG
JW4154	CTGCGCCGTTTTCCCGTTCTTTAACATCCAGTGAGAGAGACCGTAGACAGCTGCA TGCAT
JW4155	CCTGAAAGGCGAGGGCGGGTGAGTGATATCACCCGCCTGAGTCAGTGTAGGCTG GAGCTG
JW4333	GCCTTTATTCACATTCTTGC
JW4334	AATCGTCGTGGTATTCACTC
JW4337	ACTTTATCCGCCTCCATC
JW4338	ATCATGTAACTCGCCTTGAT
JW4362	GTTTTTTTGGGCTAGAGGAGATATACCATGCATCCGATGCTGAACATCG
JW4363	GCTCGAATTCGCTAGTTAACGCTTCAGAGCGTCG
JW4861	GGCTGATTTGGTTGAATG
JW4862	GGCGCATTATAGGGAGTT
JW4865	CCGCAAGGTTAAAACTCA
JW4866	CGAAGGCACATTCTCATC
JW4869	TAGTGGAAGCGTCTGGAA
JW4870	TTAGCCTTGGAGGATGGT
JW4871	ATGCAAACTGCGAATACC
JW4872	TCGTTTCCCACTTAACCA
JW4873	TCAAATCGTACCCCAAAC
JW4874	CATATCAGCGTGCCTTCT
JW4875	TCGGACATCAGGAGGTTA

JW4876	TATCCCCGGAGTACCTTT
JW4877	GCAGAAGCGGTCTGATAA
JW4878	CCTGGCAGTTCCCTACTC
JW5014	ACGACAGGTTTCCCGACT
JW5015	GTCTGATAAATTGTTAAAGAGCAGTGCCGCTTTCACAATTCCACACAACATACG
JW5017	GCTGCAAGGCGATTAAGTT
JW5068	TTACACTTTATGCTTCCGGCTCGTATGTTGTGTGGAATTGTGATAGACAGCTGCATGCAT
JW5069	GTAATCATGGTCATAGCTGTTTCCTGTGTGAAATTGTTATCCGCGTGTAGGCTGGAGCTG
JW5180	AATTGTGAAAGCGGCACTGCTCTTTAACAATTTATCAGACGCGGATAACAATTTCACACA
