# Supplementary material for: SuhB Associates with Nus Factors To Facilitate 30S Ribosome Biogenesis in Escherichia coli
Source: mBio. 2016 Mar 15;7(2):e00114-16. doi: 10.1128/mBio.00114-16 (PMC4807359; doi:10.1128/mBio.00114-16)
Supplement: Table S3 — Mutants that suppress the cold sensitivity of a ΔsuhB strain. [file mbo002162727st3.pdf]

Table S3. Mutants that suppress the cold-sensitivity of a Δ*suhB* strain.

Gene	Type of Mutation	Position Within Gene	Identification Method
rnc	Single base deletion (G)	225	PCR/sequencing
rnc	G → T substitution	634	PCR/sequencing
rnc	C → T substitution	577	PCR/sequencing
rnc	Single base insertion (A)	554	PCR/sequencing
nusE	T → A substitution	50	Whole Genome Sequencing
